# Original karst tiankeng with underground virgin forest as an inaccessible refugia originated from a degraded surface flora in Yunnan, China

**DOI:** 10.1038/s41598-022-13678-0

**Published:** 2022-06-07

**Authors:** Wei Shui, Yiping Chen, Xiaomei Jian, Cong Jiang, Qianfeng Wang, Yue Zeng, Sufeng Zhu, Pingping Guo, Hui Li

**Affiliations:** 1 College of Environment and Safety Engineering, Fuzhou University, Fuzhou, 350116 China; 2 Key Lab of Urban Environment and Health, Institute of Urban Environment, Chinese Academy of Sciences, Xiamen, 361021 China; 3 College of Urban and Environmental Sciences, Peking University, Beijing, 100871 China; 4 Chinese Research Academy of Environmental Sciences, Beijing, 100020 China

**Keywords:** Biodiversity, Plant ecology

## Abstract

Karst tiankengs are rare natural habitats, having a local microclimate different from surrounding regions. A contrast study on plant communities at the inside and outside of the primitive tiankeng was carried out by performing the qualitative analysis of species compositions of arborous and shrub layers. We found that plant communities in the primitive tiankeng belong to the subtropical moist evergreen broad-leaf forest, whereas those outside the tiankeng belong to subtropical semi-moist needle-broadleaved mixed forest. Trapped habitat of primitive karst tiankeng protects the plant communities significantly different from those in external karst ecosystems, so karst tiankeng has the great value in the plant species protection. Although the trapped habitat decreases plant species abundance inside tiankeng to some extent, highly diverse shrub species are present in the inside-tiankeng plant communities, and the primitive karst tiankeng plays an important role in the plant diversity protection. The primitive karst tiankeng is an important refugia for plant not only as a plant species protection library, but a plant diversity protection library. When implementing measures for the reduction of damages to biodiversity due to global climate changes and human activities, more attention should be paid to the primitive karst tiankeng as a small ecological refugia and biodiversity protection library.

## Introduction

Named by the Zhu Xuewen Research Team from the Karst Geology Institute of Chinese Academy of Geological Sciences in the beginning of the twenty-first century, karst tiankeng is one of the largest negative topography landscapes^1^, which is different from the doline, and is the most representative typical morphology among global cone and tower-shaped karst types^2,3^. The definition of the tiankeng has been revised several times and finally concluded that tiankeng in unified concept is a sinkhole-like negative terrain developed in a carbonate formation, leading from the ground to the surface, surrounded by craggy walls, with a depth and width in plan (top or bottom) ranging from a hundred to several hundred meters or more, and connected at the bottom to an underground river during its developmental stage^2,4^. Karst tiankeng is formed by a combination of internal and external natural forces, and its formation and evolutionary mechanisms are more particular than common karstic sinkhole^5^. However, there is no consensus among researchers as to the mechanism of its formation. Currently, the main mechanisms of tiankeng formation include: the collapse type^4^, the erosion type^4^, cave un-roofing theory^6^, hydraulic mechanism theory^7^, tiankeng evolution^8^ and depression mechanism theory^9^. According to the formation and development conditions of tiankeng, tiankeng is mainly distributed in karst areas with thick carbonate deposits, wide continuous outcrop distribution, substantial uplift of neotectonic movement, and thick vadose zone of karst aquifers^1^. Karst experts and scholars approved the rarity, significant ornamental value, scientific value^1^, world heritage value^10^, and tourism value of such karst landform formed by complicated and violent tectonic motions^11,12^. Its rare natural quality has also been approved by the United Nations Educational, Scientific and Cultural Organization (UNESCO), and many tiankeng (groups) are enlisted into the World Geopark, National Natural Heritage and the World Natural Heritage, thus attracting wide attention worldwide^13,14^.

With the 3.44 × 10^6^ km^2^ carbonatite distribution area (about 1/3 of the national territorial area), China is a country with the most extensive karst distribution and the most developed tiankeng in the world^4,5^. By 2010, of the 80 discovered tiankengs in the world, 50 were found in China. China, is titled the “King of tiankeng” and is the studying highland for karst tiankeng. However, existing studies on karst tiankeng mainly focus on the fields of geology and geomorphology, especially the classification^2,5,15–17^, geological and landform causes^8,18^, formation ages, and evolutionary mechanism of tiankeng^6,7,9^. Moreover, studies on karst tiankeng habitat and related biodiversity are few. Due to tiankeng in different parts of the world exist morphologically different and possess different names, foreign studies on biodiversity of karst topography mainly focus on dolines and sinkholes, whereas research in China are mainly focus on plant diversity^19,20^, species composition^21,22^, floral element, and succession in few accessible but highly degraded tiankengs^23^. Plant diversity in primitive tiankengs is rarely discussed.

Tiankeng is a typical and special “Continental Island.” surrounding by a trapped and steep precipice. Although its connection with the outside-tiankeng ecosystem is closer than the connection between an island and land, a trapped topography also forms a relatively closed inside-tiankeng environment^23^. Compared with tiankeng mouth, the tiankeng bottom has higher moisture and negative oxygen ionic concentration but lower temperature and thus forms a special microclimate that favorable for the animal reproduction and plant growth^24,25^. Therefore, exploring the heterogeneity of plant diversity in such special habitat holds important research significance. According to the evolution of karst tiankeng, it can be divided into primitive tiankeng, mature tiankeng, and degraded tiankeng^26^. In this paper, primary tiankeng is defined as a kind of tiankeng that are in the pre-developmental evolutionary stage with low collapse, poor accessibility, and low human disturbance. Most tiankeng in China are already degraded in various degrees, and the primitive tiankeng with vertical trapped precipices, hardly disturbed by human activities, is extremely rare (Table 1). Its local three-dimensional microclimate and microhabitat ecosystem are well preserved. Therefore, the primitive tiankeng is the most ideal region for the exploration of plant composition and structure and for plant diversity^1^. The Zhanyi tiankeng group found in Yunnan Province, where the “King of Plant” in China, exist the rare primitive tiankeng. In this area, primitive and degraded tiankengs coexist well. The subtropical moist evergreen broad-leaf forest in the tiankeng differs significantly from semi-moist evergreen broad-leaf forests in central and eastern regions of the Yunnan Province^22^. It is an ideal place to explore plant diversity and unique habitat of tiankeng.

**Table 1:** Degradation classification of karst tiankengs.

Item	Light degradation	Moderate degradation	Severe degradation	Heavy degradation
Depth-width rate	(0.45, 1]	(0.35, 0.45]	(0.1, 0.35]	(0, 0.1]
Damage degree of wall area	0–20%	21–50%	51–80%	> 81%
Quantity of rocky slope	< 1	1–2	3	> 4 (Circularity distribution)
Trapping	Good trapping	General trapping	Slightly poor trapping	Poor trapping
Pattern of pithead	Approximately ellipse	Irregular ellipse	Irregular polygon	Approximately large doline

Found in the Zhanyi tiankeng group in Yunnan Province, the “Damaosi” tiankeng is primitive tiankeng that has vertical trapped ecosystems. In this paper, we discussed whether or not the plant communities in a primitive karst tiankeng has any difference from that outside the tiankeng in the following aspects: endemic species (whether the “special phenomenon” of “Continental Island” habitat is present) and plant diversity. Qualitative analysis of species compositions in the arborous and shrub layers of the primitive “Damaosi” tiankeng and quantitative calculation of α-diversity indexes were performed. Moreover, the tiankeng is an important ecological refugia for plant species, so the aim of this paper is to determine the species refugia value and species diversity protection value of a primitive karst tiankeng^27–34^, which has well enclosure and higher isolation level with its surrounding areas. The results of this research can provide valuable scientific references for ecological restoration and local species protection in ecologically vulnerable areas in the karst and natural conservation areas in the plateau of Yunnan Province as well as other regions with similar habitat in China.

## Results

### Plant compositions inside and outside the primitive tiankeng

#### Plant composition inside the primitive tiankeng

After a rigorous plant identification, 13 arbor species, which belong to 13 genus (e.g., *Schefflera, Alangium* and *Trachycarpus*) and 12 families (e.g., *Araliaceae, Magnoliaceae* and *Alangiaceae*) in the primitive “Damaosi” tiankeng were identified. And the primitive “Damaosi” tiankeng covers 18 shrub species that belong to 16 genus (e.g., *Dalbergia, Ilex* and *Helwingia*) and 14 families (e.g., *Leguminosae, Aquifoliaceae* and *Cornaceae*) (Table 2).

**Table 2:** Floristic composition of arbor and shrub inside tiankeng.

Arbor				Shrub			
Number	Family	Genus	Species	Number	Family	Genus	Species
1	Araliaceae	Schefflera	*Schefflera heptaphylla*	1	Leguminosae	Dalbergia	*Dalbergia mimosoides*
		Trevesia	*Trevesia palmata*			Indigofera	*Indigofera tinctoria*
2	Magnoliaceae	Magnolia	*Manglietia duclouxii*			Caesalpinia	*Caesalpinia decapetala*
3	Aquifoliaceae	Ilex	*Ilex ficoidea*	2	Aquifoliaceae	Ilex	*Ilex crenata*
4	Alangiaceae	Alangium	*Alangium chinense*	3	Asparagaceae	Ruscus	*Ruscus hypoglossum*
5	Palmae	Trachycarpus	*Trachycarpus fortunei*	4	Hamamelidaceae	Distylium	*Distylium racemosum*
6	Thymelaeaceae	Edgeworthia	*Edgeworthia gardneri*	5	Moraceae	Ficus	*Ficus sarmentosa*
7	Pittosporaceae	Pittosporum	*Pittosporum brevicalyx*	6	Caesalpinioideae	Caesalpinia	*Caesalpinia sappan*
8	Rubiaceae	Adina	*Adina rubella*	7	Caprifoliaceae	Lonicera	*Lonicera ligustrina*
9	Rutaceae	Zanthoxylum	*Zanthoxylum austrosinense*	8	Araliaceae	Schefflera	*Schefflera delavayi*
10	Meliaceae	Toona	*Toona sinensis*			Nothopanax	*Metapanax delavayi*
11	Celastraceae	Euonymus	*Euonymus alatus*	9	Buxaceae	Sarcococca	*Sarcococca ruscifolia*
12	Oleaceae	Fraxinus	*Fraxinus griffithii*	10	Berberidaceae	Mahonia	*Mahonia nitens*
				11	Palmae	Chamaedorea	*Chamaedorea elegans*
				12	Lardizabalaceae	Decaisnea	*Decaisnea insignis*
				13	Theaceae	Camellia	*Camellia japonica*
				14	Betulaceae	Corylus	*Corylus yunnanensis*

#### Plant composition outside the primitive tiankeng

8 arbor species (e.g., *Cunninghamia lanceolata, Pinus yunnanensis* and *Quercus semecarpifolia*) that belong to 7 genus (e.g., *Cunninghamia, Pinus* and *Juniperus*) and 5 families (e.g., *Taxodiaceae, Pinaceae* and *Cupressaceae*), and 25 shrub species (e.g., *Myrsine africana, Cotoneaster franchetii* and *Viburnum propinquum*) that belong to 18 genus (e.g., *Myrsine, Podocarpium* and *Jasminum*) and 13 families (e.g., *Myrsinaceae, Leguminosae* and *Oleaceae*) were identified (Table 3).

**Table 3:** Floristic composition of arbor and shrub outside tiankeng.

Arbor				Shrub			
Number	Family	Genus	Species	Number	Family	Genus	Species
1	Pinaceae	Pinus	*Pinus yunnanensis*	1	Leguminosae	Podocarpium	*Hylodesmum podocarpum*
		Keteleeria	*Keteleeria evelyniana*			Campylotropis	*Campylotropis polyantha*
2	Cupressaceae	Juniperus	*Juniperus formosana*			Dalbergia	*Dalbergia mimosoides*
3	Cornaceae	Dendrobenthamia	*Cornus capitata*	2	Ericaceae	Rhododendron	*Rhododendron decorum*
4	Fagaceae	Cyclobalanopsis	*Cyclobalanopsis glaucoides*	3	Myrsinaceae	Myrsine	*Myrsine africana*
		Quercus	*Quercus variabilis*	4	Oleaceae	Jasminum	*Jasminum humile*
5	Taxodiaceae	Cunninghamia	*Cunninghamia lanceolata*	5	Coriariaceae	Coriaria	*Coriaria nepalensis*
				6	Myricaceae	Myrica	*Myrica nana*
				7	Rosaceae	Cotoneaster	*Cotoneaster franchetii*
						Photinia	*Photinia loriformis*
				8	Cornaceae	Swida	*Cornus oblonga*
				9	Anacardiaceae	Pistacia	*Pistacia weinmanniifolia*
				10	Berberidaceae	Berberis	*Berberis sargentiana*
				11	Guttiferae	Hypericum	*Hypericum henryi*
				12	Caprifoliaceae	Abelia	*Abelia grandiflora*
						Viburnum	*Viburnum congestum*
				13	Rhamnaceae	Sageretia	*Sageretia thea*
						Rhamnus	*Rhamnus parvifolia*

#### Comparison of plant compositions inside and outside the tiankeng

**Figure 1:**
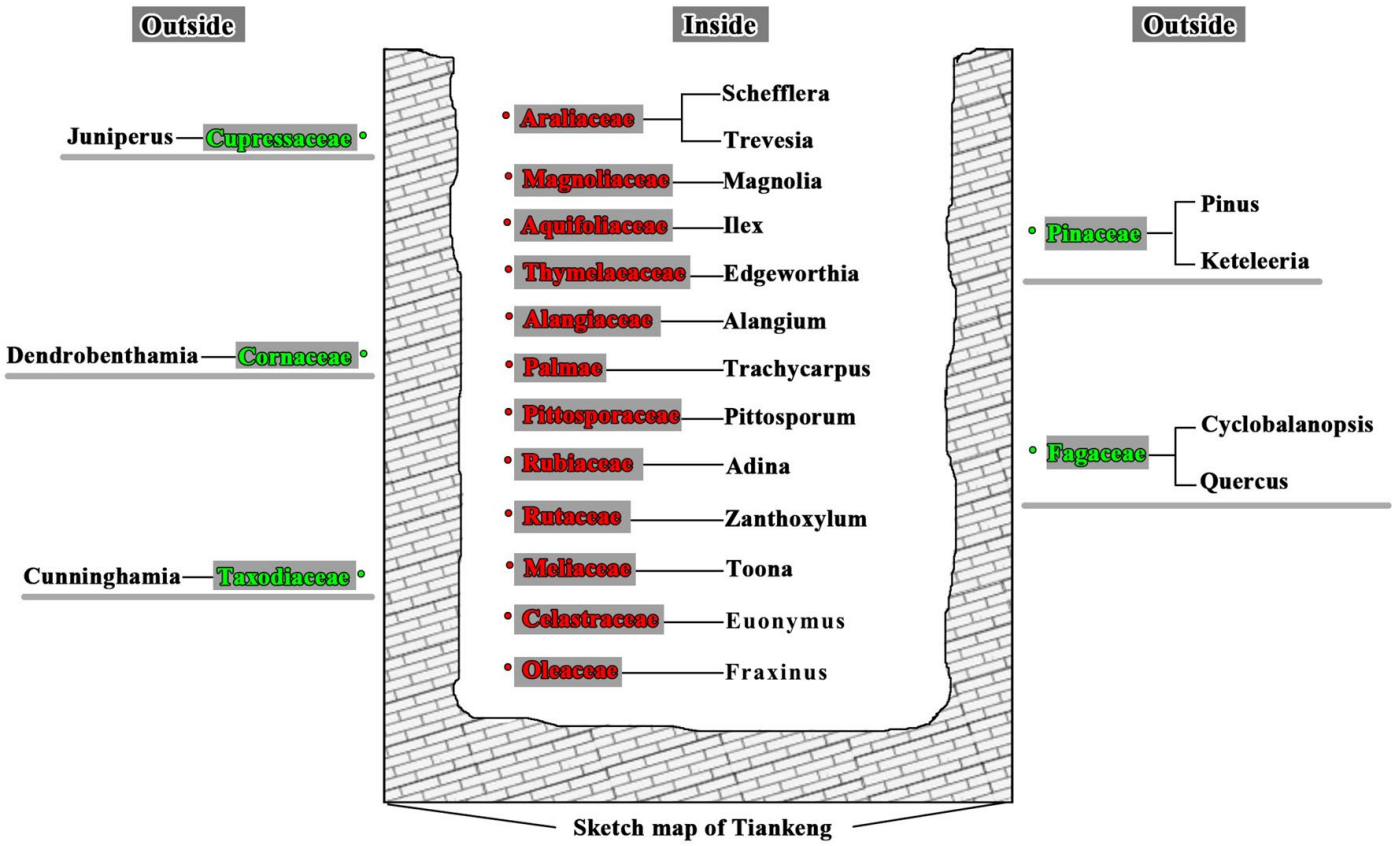
Arbor comparison between inside-tiankeng and outside-tiankeng.

**Figure 2:**
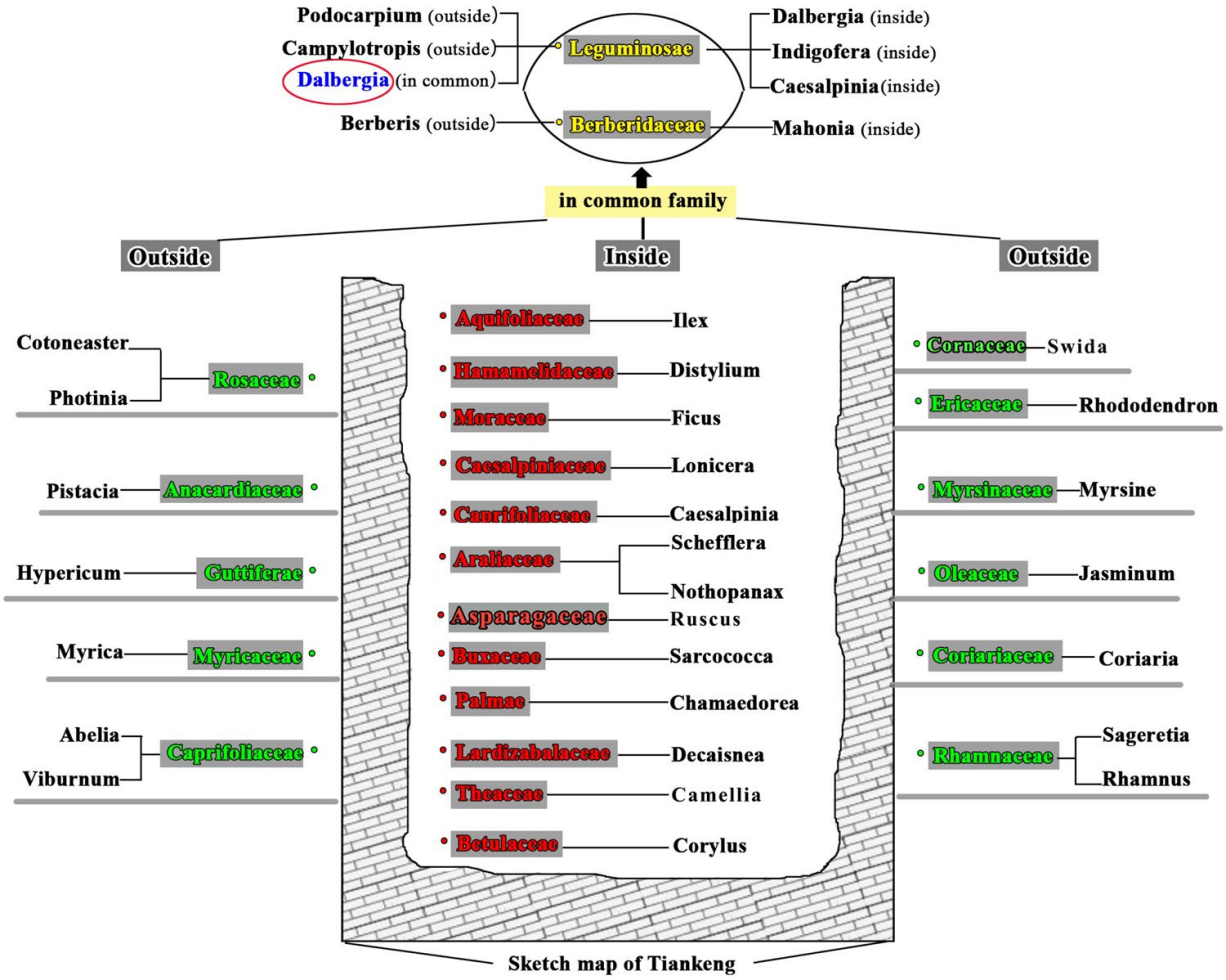
Shrub comparison between inside-tiankeng and outside-tiankeng.

For arbor species, non-overlapping significant differences were observed in “Family” between inside (5 families) and outside (12 families) the tiankeng (Fig. 1). For shrub species, 14 families inside and 13 families outside, including 3 sharing families (*Leguminosae, Cornaceae*, and *Berberidaceae)* were determined (Fig. 2). Among the 3 sharing families, only existed one shared genus (*Dalbergia*). In other words, significant plant composition differences existed inside and outside the primitive “Damaosi” tiankeng. Inside-tiankeng plant communities, we found that moist evergreen broad-leaf forest species, such as *Manglietia fordiana,* are dominant in the arborous and shrub layers, and pteridophytes, such as *Allantodia viridissima,* in the moist evergreen broad-leaf forest are major species in the herbaceous layer. Outside-tiankeng plant communities, semi-moist evergreen broad-leaf forest species, such as *Evelynia keteleevia,* is the major species according to sample plot survey.

**Figure 3:**
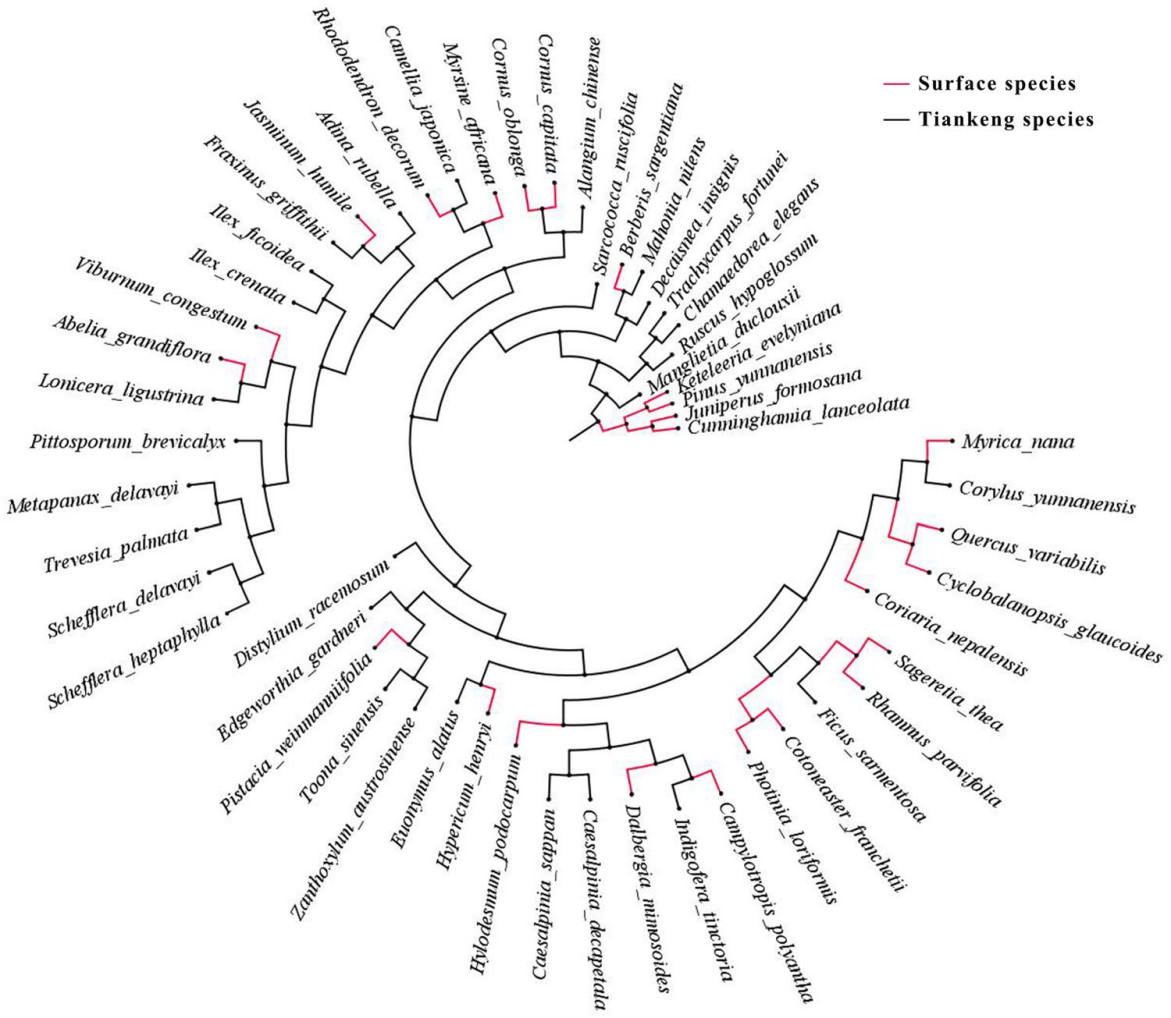
The evolutionary tree between inside-tiankeng and outside-tiankeng species.

According to the evolutionary tree of plant species in the tiankeng of Damaosi (Fig. 3), we found seven groups of species from both inside and outside the tiankeng directly derived from the same evolutionary clade: *Euonymus alatus* & *Hypericum henryi*, *Indigofera tinctoria* & *Campylotropis polyantha*, *Fraxinus griffithii* & *Jasminum humile*, *Lonicera ligustrina* & *Abelia grandiflora*, *Mahonia nitens* & *Berberis sargentiana*, *Corylus yunnanensis* & *Myrica nana* and *Camellia japonica* & *Rhododendron decorum*. Meanwhile, it also showed that there was a certain genetic relationship between the species inside and outside the tiankeng.

### Plant diversity inside and outside the primitive tiankeng

#### α-Diversity index of plant communities inside the tiankeng

Plant diversity inside the tiankeng was investigated by sampling the entire site. Data collections were performed by the adjacent gridding method. The α-diversity index of each sample plot was calculated, and the mean was used as the α-diversity of the entire plant communities at the bottom. Based on the means of α-diversity index, the test results of Margalef abundance, Shannon–Wiener diversity, Pielou evenness, and Simpson dominance in the arborous and shrub layers at the bottom were generally consistent (Table 4). Statistical differences ranged from 23.3 to 34.4% in the arborous layer, and 30.4 to 52.1% in the shrub layer. For both arborous and shrub layers, obvious differences of abundance, diversity, and species composition were observed at different bottom positions, reflecting certain spatial heterogeneity of illumination, soil, humidity, and wind speed at different bottom positions.

**Table 4:** Diversity of woody layer inside tiankeng.

Index	Margalef		Shannon–Wiener		Pielou		Simpson	
	S	A	S	A	S	A	S	A
Average	1.179	1.101	1.023	0.997	1.706	1.697	0.537	0.531
Standard deviation	0.614	0.372	0.433	0.343	0.519	0.395	0.209	0.168
Coefficient of variation	0.521	0.338	0.423	0.344	0.304	0.233	0.389	0.317

S showed shrub layer; A showed arbor layer.

#### α-Diversity index of plant communities outside the tiankeng

**Figure 4:**
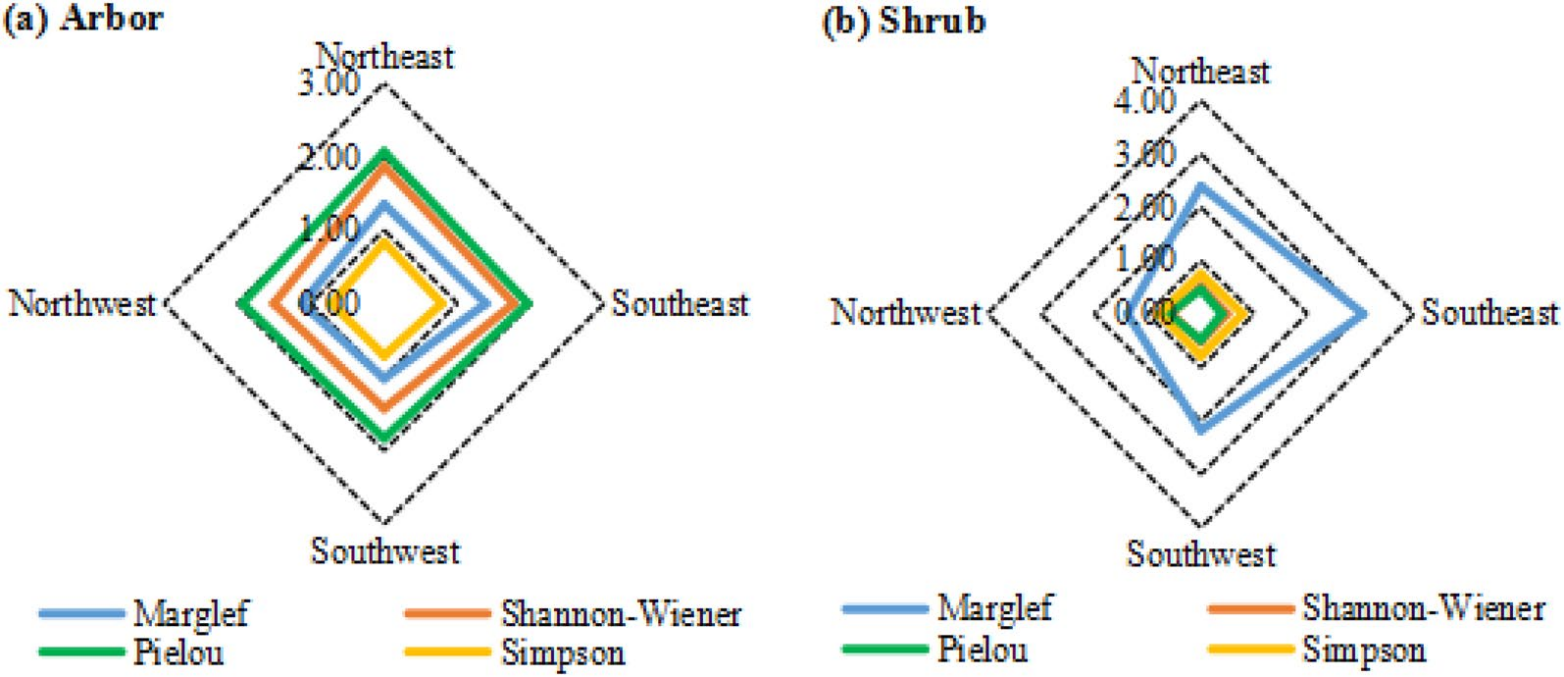
Diversity distribution of different directions outside tiankeng.

Four sample plots were set outside the primitive “Damaosi” tiankeng to investigate plant diversity. Margalef abundance, Shannon–Wiener diversity, Pielou evenness, and Simpson dominance of the arborous layer in southwestern, northwestern, southeastern, and northeastern parts had minimal differences (Fig. 4). The variable coefficients of the indexes in all directions only ranged from 5.0 to 14.7%, indicating that the arborous layer is relatively uniform with respect to species abundance, diversity, and species composition in a trapped topography.

Shannon–Wiener diversity, Pielou evenness, and Simpson dominance of the shrub layer on four directions were relatively uniform, the variable coefficient ranged from 2.5 to 19.1%. However, Margalef abundance varied dramatically, and the variable coefficient reached as high as 30.6%. The extent of variation on the value of Margalef abundance was in the following order: southeast > northeast > southwest > northwest. In other words, the plant abundance of the shrub layer in the eastern part outside the tiankeng was higher than that in the western part, and the plant abundance of the shrub layer in the southern part was higher than that in the northern part.

Based on comparison of α-diversity index between the arborous and shrub layers outside the tiankeng, the shrub layer showed higher plant abundance (S = 2.246 > A = 1.218), and the arborous layer showed higher species diversity (S = 0.523 < A = 1.639) (Table 5).

**Table 5:** Diversity distribution of woody layer outside tiankeng.

Samples	Marglef		Shannon–Wiener		Pielou		Simpson	
	S	A	S	A	S	A	S	A
Southwest	2.173	1.016	0.523	1.423	0.485	1.828	0.780	0.706
Northwest	1.378	1.120	0.555	1.502	0.614	1.930	0.785	0.716
Northeast	2.391	1.349	0.522	1.865	0.456	2.065	0.745	0.832
Southeast	3.045	1.386	0.494	1.766	0.393	1.955	0.785	0.786
Average	2.246	1.218	0.523	1.639	0.487	1.945	0.774	0.760
Standard deviation	0.688	0.179	0.025	0.210	0.093	0.097	0.019	0.060
Coefficient of variation	0.306	0.147	0.048	0.128	0.191	0.050	0.025	0.078

S showed shrub layer; A showed arbor layer.

#### Comparison between the α-diversity indexes obtained inside and outside the tiankeng

**Figure 5:**
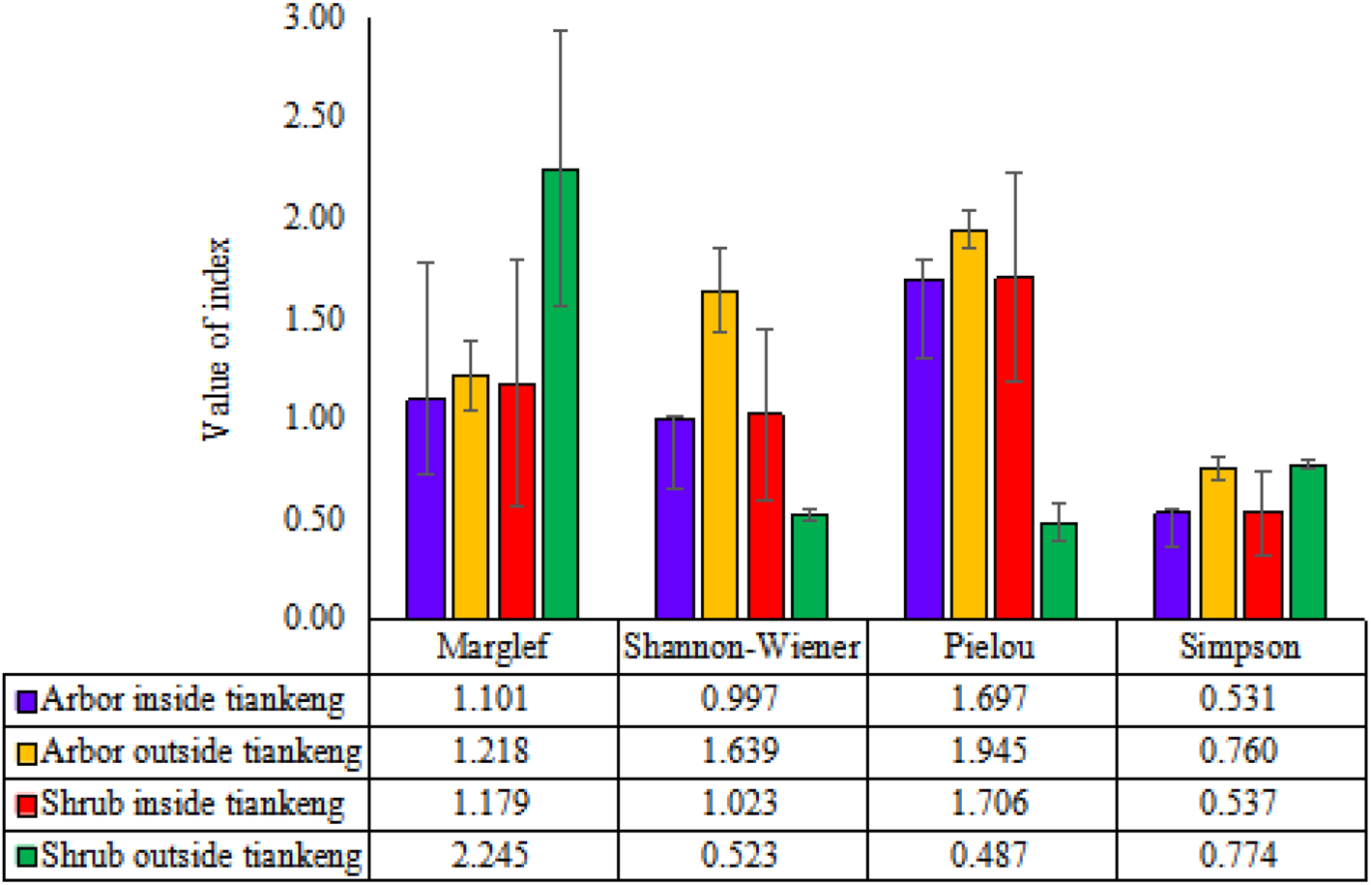
Community diversity comparison between inside tiankeng and outside tiankeng.

With respect to arborous species, the Margalef abundance, Shannon–Wiener diversity, Pielou evenness, and Simpson dominance outside the tiankeng were all higher than inside. A great gap between the areas outside and inside the tiankeng with respect to species abundance was observed. These results reflect that the arborous species diversity in areas outside the tiankeng is higher than that inside. For shrub species, Shannon–Wiener diversity and Pielou evenness were higher in areas inside than those outside, whereas Margalef abundance and Simpson dominance showed the opposite result. In summary, higher arborous species diversity was observed outside the primitive “Damaosi” tiankeng, but higher shrub species diversity was observed inside it (Fig. 5).

## Discussion

The theory of island biogeography proposed by MacArthur and Wilson in 1967 is one of the most important theories in ecological study^35^, and provides important theoretical references for biodiversity protection. It was initially employed to study biodiversity in ocean islands, then gradually used to study biodiversity in “Continental islands”, such as high mountains, natural conservation areas, lakes, and forests with surrounding open areas^36,37^. Naturally occurring habitat fragmentation in the karst region is prominent^38^. Raschmanová^34^ and his research team showed that, in karst geomorphological landscapes, dark, wet, and cold habitats in a doline are similar to a typical island habitat in temperate areas found in the middle of Europe. The special internal microclimate in karst tiankeng with good trapping and high vertical depth showed obvious “island” characteristics with surrounding regions. Culver studied karst environment and found that the vertical topography in karst landscape can intercept extreme climate events to some extent and can lower extreme temperature significantly in the local area. This conclusion strongly supports the “island” characteristic of karst tiankeng^39^. Moreover, such “island” habitat provides an important place for survival and reproduction of animals and plants, and especially can become the ecological refugia of cold-resistant plants under climate change^27–32^.

Daily showed that the changing land utilization pattern causes landscape fragmentation and ecosystem simplification and separates biotic populations from communities, resulting in the sharp reduction of global animal and plant diversity^40^. Although the topography in tiankeng causes local habitat fragmentation, the vertical steep precipices and trapped morphology fail to make the primitive “Damaosi” tiankeng an isolated “island” with poor ecological environment. On the contrary, the primitive “Damaosi” tiankeng possesses abundant plant species and the vegetation coverage reaches about over 95%. Arbor and shrub species inside and outside differ significantly. The vegetation types in the area of tiankeng group are mainly dominated by subtropical semi-moist needle-broadleaved mixed forest, whereas the dominant vegetation types inside the primitive “Damaosi” tiankeng are moist evergreen broad-leaved ones in the subtropical evergreen broad-leaved forest, which mainly occur in moist monsoon climate regions and its development would be restricted in the middle and east plateaus of Yunnan^24^. However, protected by primitive “Damaosi” karst tiankeng’s natural precipices, the native vegetation types were maintained, and the plant communities are resistant to the effects of climate change and human activities. Bartgis^41^ discovered some plant species that are rare or endangered in local areas could be found in sinkhole pond. Yu^42^ and her team studied *Xylophyta* diversity in the ecological niche in the Stone Forest Geopark in Yunnan Province, and found that ecological niches like crack ditches, deep karst pit, sphenoid trough, and corrosion clitter still maintain some local plant propagation after suffering from strong human disturbances, whereas local plant propagation of other ecological niches are absent. Dang^43^ studied the origins and evolution of flora in 14 tiankengs in the Guangxi Dashiwei tiankeng group and discovered 1 new genus and 5 new species. Our results on plant communities in the primitive tiankeng also proved that a karst tiankeng formed by long-time tectonic motions is not only a kind of unique and large topography but also a precious species protection library. It is an important material to study vegetation evolution in tiankeng areas and an important plant species protection library.

Climate change and habitat loss cause serious threats to global biodiversity and ecosystem. Protecting habitat integrity and species refugia is crucial to wild animals and plants. Climate change has caused the migration of plants on biotic formation. Thus, more attention must be paid not only to existing large refugia but also to small refugia in some landscape areas and small natural conservation areas. Tiankeng is one of rare natural habitats, and its unique habitats are precious refugia for plant species and diversity^44^. This species sanctuary function has important value on cope with future climate changes and vulnerable ecological environment in karst regions, thus karst tiankengs are important protected objects of small ecological refugia.

Diversity of microenvironment in one community environment is positively correlated with community abundance. Compared with outside-tiankeng habitat, inside-tiankeng habitat are limited and local environmental heterogeneity is weak. Thus, the inside-tiankeng microenvironmental diversity is lower than outside-tiankeng. Moreover, due to the attributes of depth and vertical trapped precipice, internal illumination, temperature, wind speed, and other environmental resources are inferior in the inside, resulting in low species abundance inside the tiankeng. Meanwhile, the “intermediate disturbance hypothesis” suggests that certain disturbances from human beings or nature contribute to species diversity growth. Connell and Lawton^45^ disclosed that species abundance is related with disturbances to the crown canopy. Species abundance decreases upon strong interferences to forest stand, and forest community is unable to reach the mature forest form. Possibly, few species are present in slightly disturbed forests, but the species abundance of forest increases to some extent after moderate disturbance. Helmus^46^ and his team took the chameleon population on the Caribbean Islands as the example and found that biodiversity on islands is sensitive to human disturbance except for separation degree and island areas. The immigration of external species due to human activities increases biodiversity on islands, and thus human activities considerably affect biodiversity and abundance in habitats in isolated islands. “Damaosi” tiankeng is primitive, and its internal ecosystem has been slightly disturbed by human activities. And the tiankeng group is in the Haifeng Natural Conservation Area where human activity is limited, so the outside-tiankeng plant community suffer slight or intermediate disturbances. As a result, the low species abundance inside the tiankeng might conform to the “intermediate disturbance hypothesis.”

According to comparison of species abundance outside the primitive “Damaosi” tiankeng on different directions, the shrub abundance in the east is higher than that in the west, and the shrub abundance in the south is higher than that in the north. The results of the field investigation showed that the primitive “Damaosi” tiankeng is located on the inclined concave slope surface of groove head of the small river basin and marginal terrains and are characteristic of “high in the north and low in the south”. And the “Damaosi” tiankeng has different soil corrosion characteristics. The greatest number of flat terrains and highest frequency of soil accumulation are observed in the southeast. Slope is the main terrain factor that influences the surface distribution of vegetation^47^, such as grass, vine, and shrubs. Moreover, soil accumulation is the key condition for vegetation growth and plant community succession in the south karst region^48,49^. Fewer limestone, more soil accumulations, and better water retention capacity are present in the east part of outside tiankeng compared to west. Thus, the former is more favorable for plant growth and has higher species abundance. Du^50^ discussed the coupling relationship between plant diversity and soil properties in a karst vulnerable ecosystem, and they found that soil minerals and microorganisms both regulate vegetation composition, community type, and growth conditions. Conversely, soil properties also can change with vegetation. Therefore, vegetation plays an important role in ecological recovery, conservation of water and soil, and soil accumulation in karst regions with serious stony desertification problems in China.

According to our results, arbor species diversity is higher outside the primitive “Damaosi” tiankeng, but shrub species diversity is higher inside. Illumination is an essential factor for plant growth and photosynthesis. The vertical precipices and trapped topography in karst tiankeng block illumination, and shrub species possess higher survival advantages in such “underground forest” than arborous species. Studies on plant diversity of different ecosystems in karst fengcong depression disclosed that maximum species diversity occurs in secondary forests. The primitive “Damaosi” tiankeng has a primary forest inside it and a secondary forest outside. Although trapped habitat conditions decrease plant abundance to some extent, shrub species diversity inside the tiankeng is still higher than that in the outside, thus, the unique habitat of karst tiankeng is an important plant diversity protection library. Dolinar^33^ studied the flora of vascular plants in some phytogeographic regions in Solvenian, and concluded that the abundant plant diversity in this region is mainly attributed to the protection of karst ponors. Shui^8^ and his team discovered that plant diversity in the habitats of karst tiankeng is different from common karst topographies such as dolines and sinkholes. Liu^51^ and his team also pointed out that tiankeng has higher plant abundance than other karst depression. Many studies reported that karst dolines are important species diversity protection libraries^27,28,33^. A karst tiankeng, which has larger size and higher closure and higher isolation degree with its surrounding areas, is believed to possess an exceptional value in species diversity protection. The karst region in south China is not only an important basis for biodiversity protection but also an important habitat for rare and endangered animals and plants. It possesses significant world heritage value to protect biodiversity. Meanwhile, the role of karst tiankeng as a small biodiversity protection library should be emphasized further.

## Methods

### Study area

**Figure 6:**
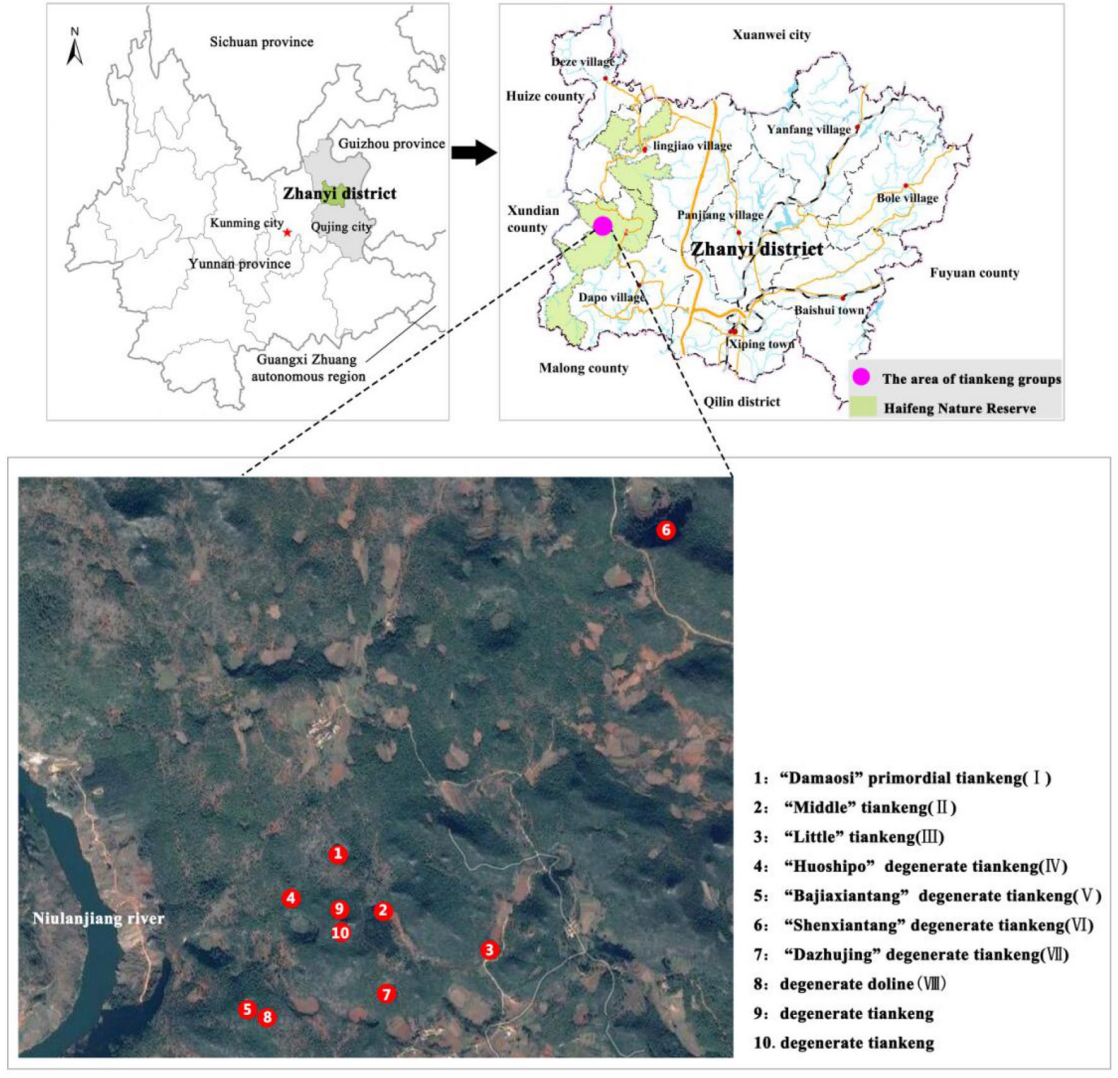
Location of Zhanyi karst tiankeng group in Yunnan Province. The map at the top is from the ArcGIS 10.2 software (https://www.esri.com) by vector processing based on the administrative map. The map below is from remote sensing image obtained by United States Geological Survey (USGU, https://www.usgs.gov/), and preprocessed by ENVI 5.0 software (http://www.harrisgeospatial.com). These maps were merged by the Photoshop CS6 software (http://www.adobe.com) and marked the names of these tiankengs.

The Zhanyi tiankeng group lies in the Dapo Town within the Haifeng Natural Preservation Area in the Yunnan Province. This area belongs to the Jinsha river system and control area of the Niulan River Basin, which is the first-level tributary of Jinsha river (Fig. 6). It has a typical subtropical plateau monsoon climate, having windy weather and drought in winter and spring; wet, warm, and rainy weather conditions in summer and autumn; and small annual temperature differences and large daily temperature differences^52^. This climate lays an important basis for the formation of abundant plant diversity.

**Figure 7:**
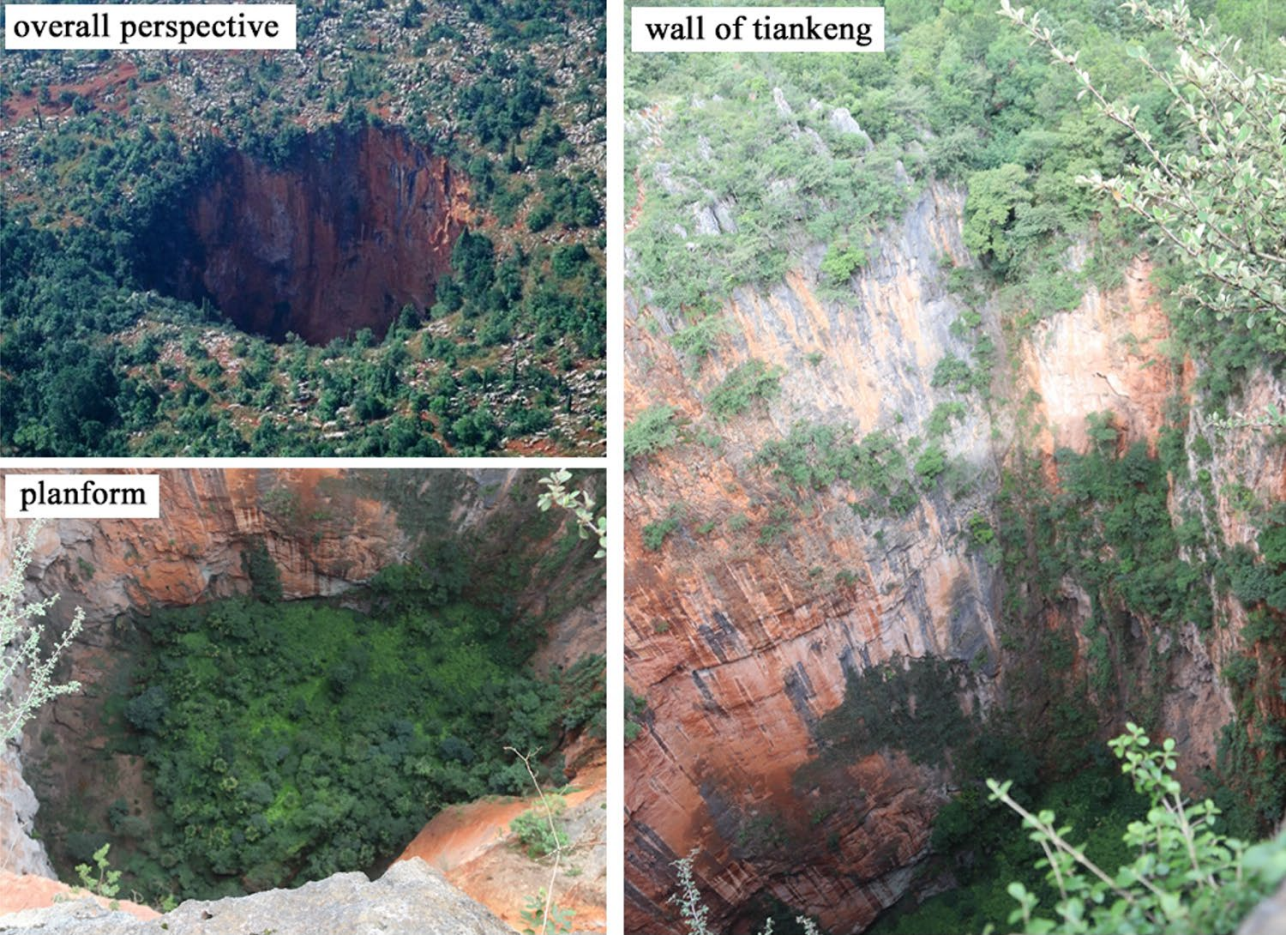
Panorama and profile map of Damaosi.

On August 2016, the research team made a field investigation in Zhanyi tiankeng group in Yunnan Province. Ten tiankengs were investigated, and the basic data of some tiankengs were collected (Table 6). Tiankeng I (“Damaosi”) is a primitive tiankeng that has vertical precipices and get few human disturbances. According to official measurement, its maximum diameter can reach approximately 200 m, and in this survey, the longest diameter measured was 136.8 m. Tiankeng II and Tiankeng III which have slightly disturbed by human activities, and is called “middle tiankeng” and “small tiankeng,” respectively. Tiankengs IV, V, and VI are moderately degraded tiankengs and are called by locals as “Huoshipo,” “Bajiaxiantang,” and “Shenxiantang,” respectively. Previously, the bottom of these degraded tiankengs had been used for agriculture. Currently, the farmlands reverted to woodlands, and vegetation recovered to some extent. Tiankeng VII is a seriously degraded tiankeng and is called by locals as “Dazhujing”, it is large and have a maximum diameter of about 455 m. Its bottom contains one blind river and several karst caves. Tiankeng VIII is a degraded doline and is in the west of “Bajiaxiantang”. In this paper, the primitive “Damaosi” tiankeng, which has hardly influenced by human activities, was chosen as the research object to study the tiankeng’s plant diversity (Fig. 7).

**Table 6:** Basic information of karst tiankengs and dolines (m), “a” showed the elevation data measured in the edge of tiankeng/dolines; “b” showed the elevation data measured in the bottom of tiankeng/dolines.

Mark	Tiankeng/doline	Location		Elevation	Long diameter	Short diameter	Depth
		Latitude (N)	Longitude (E)				
I	Damaosi	25° 47′ 19.9″	103° 33′ 55.5″	2024–2036^a^	136.8	76.6	186.7
II	Middle tiankeng	25° 47′ 15.6″	103° 34′ 2.7″	1996–2000^a^	62.0	50.0	153.0
III	Little tiankeng	25° 47′ 59.4″	103° 34′ 21.0″	1945–1950^a^	75.0	72.0	179.2
IV	Huoshipo	25° 47′ 10.1″	103° 33′ 50.9″	1961–1965^a^	150.0	132.0	64.4
V	Bajiaxiantang	25° 47′ 6.4″	103° 33′ 40.7″	2012–2015^b^	240.0	197.7	69.8
VI	Shenxiantang	25° 48′ 11.2″	103° 34′ 45.8″	2028–2031^b^	421.9	348.7	148.7
VII	Dszhujing	25° 46′ 51.4″	103° 34′ 27.0″	1901–1907^a^	455.6	365.0	123.6
VIII	–	25° 47′ 6.1″	103° 33′ 45.8″	1971–1975^b^	125.0	–	–

### Sample design

**Figure 8:**
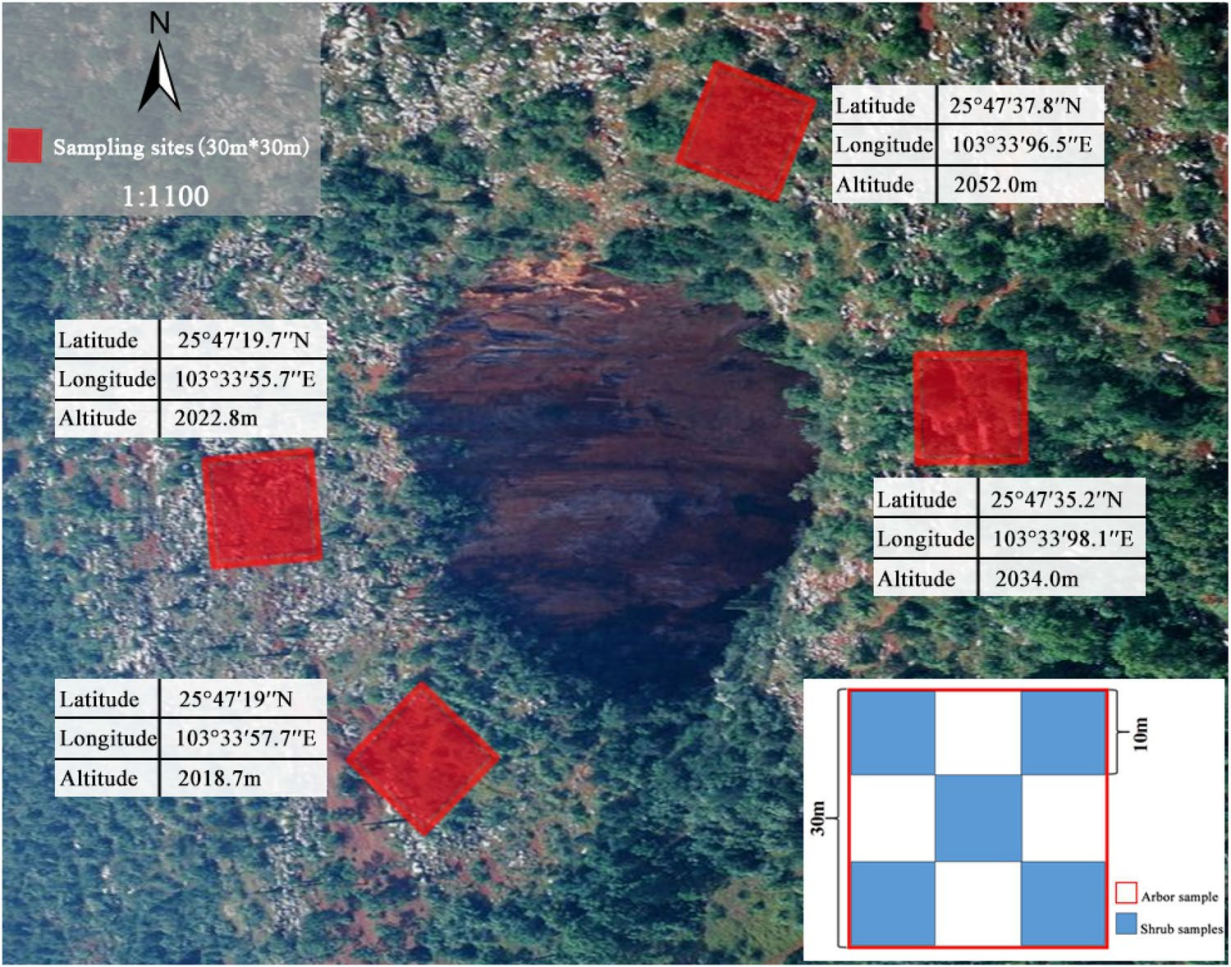
Plant community sample setting outside tiankeng.

**Figure 9:**
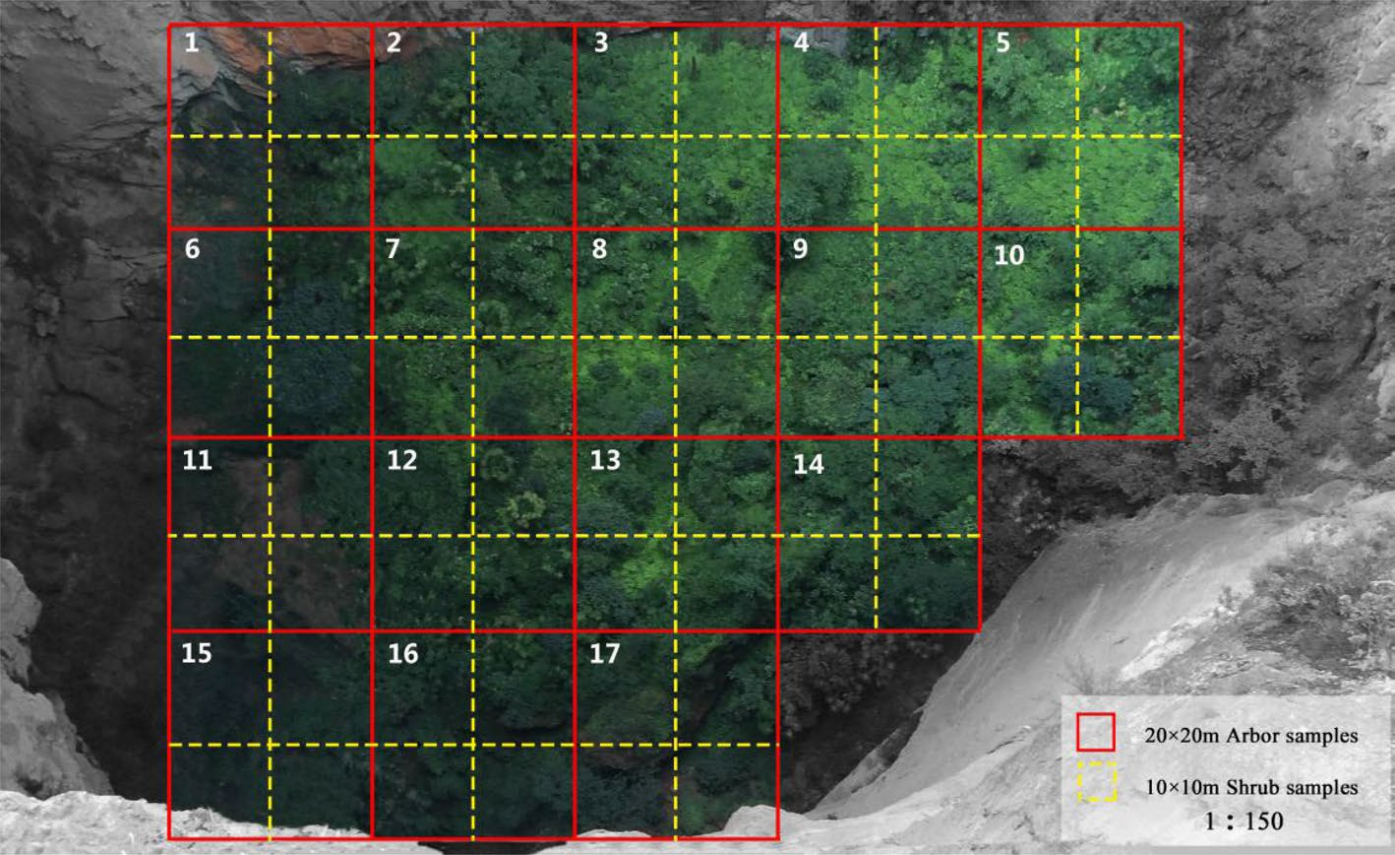
Plant community sample setting inside tiankeng.

30 m × 30 m sample plot was established for the census of community at the outside-tiankeng habitat, and these quadrats was distributed in the southwestern, northwestern, southeastern, and northeastern parts of the primitive tiankeng, respectively (Fig. 8). In each sample plot, five 10 m × 10 m shrub sample sites were set in a “pentalobe” pattern. The arborous layer was studied by a full sample survey (Fig. 9). The bottom area of “Damaosi” tiankeng was approximately 0.8 × 10^4^ m^2^. Plant species at the bottom were investigated. For the convenience of data statistics, 17 sample sites (20 m × 20 m) were set by the adjacent gridding method, and plant species in each site were investigated. Given the poor accessibility of a primitive tiankeng, an unmanned aerial vehicle and high-resolution photographs technique were used to collect data from the bottom for plant species survey. We then mainly focused and analyzed on the arborous and shrub layers.

### Identification of species

With field sample plot setting, species survey and data statistics, we performed the preliminary field species identification, and unidentified species were identified in the laboratory on the basis of *Flora of China*, *Yunnan Local Flora*, “*China Herbarium*” “*China Plant Species Database*” and “*PPBC China Plant Image Library*”. Those remained unidentified were subjected to further consultations from experts for three times. Two consultations were performed online, one of which was performed by holding an expert consultation conference. In this conference, unidentified plant species were presented to experts in phytological and ecological fields. After the conference, the opinions of the experts were evaluated and summarized to obtain useful data.

Consultants who are expert in plant species identification were mainly selected by two ways. First, experts in related fields were consulted. Second, experts were assessed by reading his articles. A total of 22 expert testimonies were sent, and 15 of which were collected effectively. Plant species identification received strong support from 10 senior experts and 5 scholars with PhD, all of them have the outstanding achievements in bioscience and ecology fields.

### Data analysis

#### Evolutionary tree construction

The Latin name lists of sample species were entered into the Phylomatic platform, and constructing the species evolutionary trees by its database, which is based on the APG III, it can automatically constructing the phylogenetic topology of species, integrating the evolutionary tree information according to the slik algorithm, and outputting the evolutionary trees with branch lengths.

#### Margalef abundance

Margalef abundance refers to the quantity of species in one community or environment. It is an index that reflects species abundance in a biocommunity (sample).

1 $$ D = \left( {S - 1} \right)/\ln N $$

where D is the Margelef richness index; S is the number of species; N is the total number of individuals.

#### Shannon–Wiener diversity

Shannon–Wiener diversity, which is also known as information index, is used to study heterogeneity. In this index, the greater the number of species in one community, the higher the uncertainty of the classification of one random individual and the higher the diversity are^53^.

2 $$ H^{\prime }= - \sum\limits_{i = 1}^{s} {(P_{i} } \ln P_{i} ) $$

where H′ is Shannon–Wiener diversity index; P_i_ is the relative abundance of the ith species, calculated as P_i_ = n_i_/N.

#### Pielou evenness

Pielou evenness reflects the uniformity of a surveyed community. Pielou defined it as the ratio between measured diversity (H′) and the maximum diversity (H′max, the diversity of completely even community under the given number of species S) in 1977^54^. In this paper, it was derived from the Shannon–Wiener diversity index.

3 $$ J = - \sum\limits_{i = 1}^{s} {\left( {P_{i} \ln P_{i} } \right)} /\ln S $$

where J is Pielou evenness index; S is the number of species.

#### Simpson dominance

Simpson dominance is one of the most famous and the earliest dominance indexes. It mainly measures concentration of studied communication, which is opposite to the diversity index^55^.

4 $$ H = 1 - \sum\limits_{i = 1}^{S} {\mathop {(N_{i} /N)}\nolimits^{2} } $$

where H is Simpson dominance, P_i_ is the relative abundance of the species, calculated as P_i_ = n_i_/N.

### Ethical statement

The authors declare that this manuscript comply with the IUCN Policy Statement on Research Involving Species at Risk of Extinction and the Convention on the Trade in Endangered Species of Wild Fauna and Flora.

## Data Availability

The datasets generated during the current study are available from the corresponding author on reasonable request.
